# Acalabrutinib in High‐Risk Chronic Lymphocytic Leukaemia Naïve Patients: An Italian Multicenter Retrospective Observational Real‐Life Experience

**DOI:** 10.1002/hon.70033

**Published:** 2025-01-14

**Authors:** Idanna Innocenti, Annamaria Tomasso, Diana Giannarelli, Lydia Scarfò, Roberta Murru, Andrea Visentin, Anna Maria Frustaci, Francesca Morelli, Candida Vitale, Antonio Mosca, Alessandro Sanna, Giuliana Farina, Roberta Laureana, Massimo Gentile, Andrea Galitzia, Raffaella Pasquale, Francesco Autore, Jacopo Olivieri, Azzurra Romeo, Marina Deodato, Luca Stirparo, Andrea Corbingi, Vanessa Innao, Francesca Perutelli, Francesca Martini, Paolo Sportoletti, Alberto Fresa, Maria Ilaria Del Principe, Alessandra Tedeschi, Marta Coscia, Paolo Ghia, Luca Laurenti

**Affiliations:** ^1^ Department of Laboratory and Haematological Sciences Haematology Area Fondazione Policlinico Universitario A. Gemelli IRCCS Università Cattolica del Sacro Cuore Roma Italy; ^2^ Hematology Section Department of Radiological and Hematological Sciences Università Cattolica del Sacro Cuore Roma Italy; ^3^ Facility of Epidemiology and Biostatistics GSTeP Fondazione Policlinico Universitario Agostino Gemelli IRCCS Roma Italy; ^4^ Strategic Research Program on CLL IRCSS San Raffaele Milano Italy; ^5^ Medical School Università Vita Salute San Raffaele Milano Italy; ^6^ Hematology and Stem Cell Transplantation Unit Ospedale Oncologico A. Businco ARNAS G. Brotzu Cagliari Italy; ^7^ Hematology Unit Department of Medicine—DIMED University of Padua Padova Italy; ^8^ Department of Hematology Niguarda Cancer Center ASST Grande Ospedale Metropolitano Niguarda Milano Italy; ^9^ Department of Experimental and Clinical Medicine University of Florence Firenze Italy; ^10^ Department of Molecular Biotechnology and Health Sciences University of Torino and Division of Hematology University Hospital (A.O.U.) Città della Salute e della Scienza di Torino Torino Italy; ^11^ Hematology Department of Oncology AOU Careggi Firenze Italy; ^12^ Hematology and Medical Oncology AORN Sant’Anna e San Sebastiano Caserta Italy; ^13^ Hematology Department of Biomedicine and Prevention Tor Vergata University of Rome Roma Italy; ^14^ Hematology Unit AO of Cosenza Cosenza Italy; ^15^ Department of Pharmacy Health and Nutritional Sciences University of Calabria Rende Italy; ^16^ Department of Medical Sciences and Public Health University of Cagliari Cagliari Italy; ^17^ SOC Clinica Ematologia Azienda Sanitaria Universitaria Friuli Centrale (ASU FC) Udine Italy; ^18^ Hematology and Stem Cell Transplantation Unit S.M. Goretti Hospital Polo Universitario Pontino Latina Italy; ^19^ UOC di Ematologia Azienda Ospedaliera di rilievo nazionale di alta specializzazione ARNAS—Garibaldi di Catania Catania Italy; ^20^ Department of Medicine and Surgery Institute of Hematology Centro di Ricerca Emato Oncologica (CREO) University of Perugia Perugia Italy; ^21^ Hematology‐Oncology and Stem‐Cell Transplantation Unit Department of Onco‐Hematology and Innovative Diagnostics Istituto Nazionale Tumori‐IRCCS‐Fondazione G. Pascale Napoli Italy; ^22^ Department of Medicine and Surgery University of Insubria, and Hematology Division ASST Sette Laghi Ospedale di Circolo Varese Italy

**Keywords:** acalabrutinib, cBTKi, CLL, high risk

## Peer Review

The peer review history for this article is available at https://www.webofscience.com/api/gateway/wos/peer-review/10.1002/hon.70033.

**FIGURE 1:**
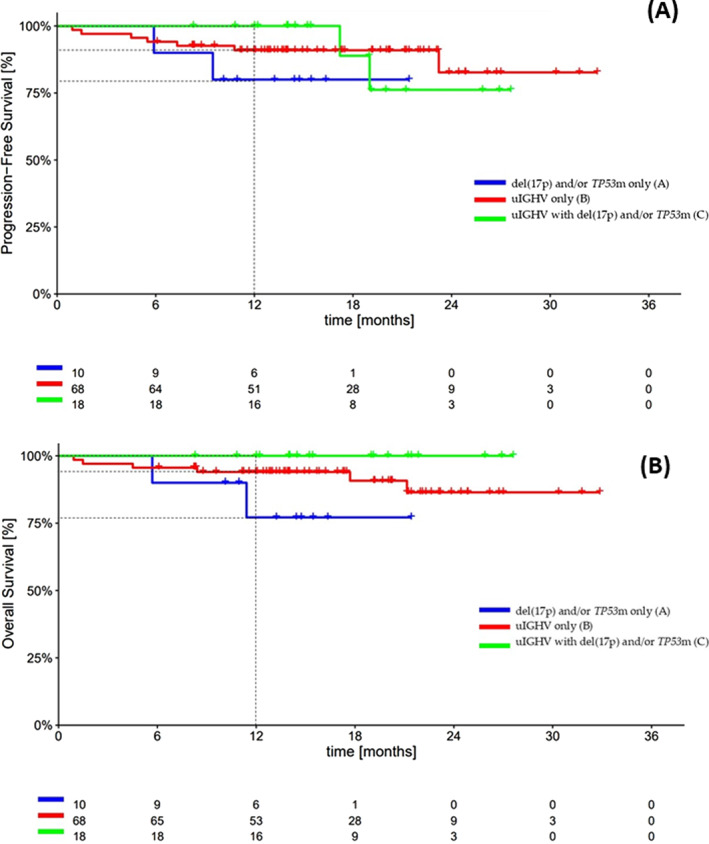
(A): Kaplan–Meier curves of PFS in TN patients with high‐risk features. (B): Kaplan–Meier curves of OS in TN patients with high‐risk features.

To the Editor,

In recent years, the treatment of chronic lymphocytic leukaemia (CLL) has changed considerably, in favour of a chmo‐free approach with covalent BTK inhibitors (cBTKis), such as ibrutinib, acalabrutinib and zanubrutinib, that have improved therapeutic results even in patients with an unfavourable risk profile [del(17p), *TP53*m, unmutated IGHV genes (uIGHV)] [1, 2, 3, 4]. The ‘continuous therapy’ with cBTKis achieves a high response rate, predominantly partial, with an excellent progression free survival (PFS) and overall survival (OS) even in the setting of high‐risk (HR) patients [5, 6]. ESMO Guideline update on new targeted therapies recommends the use of BTKi as first line for patients with a *TP53*m and/or del(17p) [7], because reported PFS rates suggest longer duration of disease control, and for patients with an uIGHV without a *TP53*m or del(17p), especially if unfit or elderly. Despite these data, in clinical practice it's still unclear whether there's a difference of efficacy of cBTKis in HR subgroups [del(17p), *TP53*m, uIGHV]. Therefore, this nationwide multicentre retrospective study aims to describe efficacy and safety of acalabrutinib in TN patients with HR CLL and to identify which subgroup of HR may benefit most from this treatment. This study was conducted according to the Helsinki Declaration, Good Clinical Practice and the applicable national regulations, and approved by the local Ethical Committee (study number 6390). Its primary endpoint was to evaluate the efficacy of acalabrutinib as first‐line treatment in patients with CLL with an unfavourable biological profile [del(17p) and/or *TP53*m and/or uIGHV] in terms of overall response rate (ORR). The secondary endpoints were to compare PFS and OS across subgroups of HR CLL treated with frontline acalabrutinib and to assess its tolerability profile. We enrolled 98 TN patients with HR CLL, who started acalabrutinib between June 2021 and June 2023. Response evaluation was done at 12 months since the start of treatment [8]. Data regarding definitive treatment discontinuation and safety were also collected. We analysed the survival data by stratifying patients into further subgroups according to the HR biological characteristics [del(17p) and/or *TP53*m only, with uIGHV only, uIGHV with del(17p) and/or *TP53*m]. Two patients were not analyzed due to unavailability of all biological data. Clinical characteristics and biological features at baseline are reported in Table 1. The 10% of patients had del(17p) and/or *TP53*m only (group A), 71% uIGHV only (group B) and 19% had uIGHV with del(17p) and/or *TP53*m (group C). After a median follow up of treatment of 16 months, the 86% of patients remained on treatment. ORR among all patients at 12 months was 94% as follows: complete remission (CR) and partial remission (PR) rate were 17% and 77%, respectively. ORR at 12 months were 80%, 95% and 93% in group A, group B and group C, respectively (Table 1). The 12 months PFS and the 24‐months estimated PFS were 92% and 81%, and they were similar between the three subgroups of patients according to their biological risk. At 12 months, PFS were 80% in group A, 91% in group B and 100% in group C (*P* = 0.45) (Figure 1). The 12‐months OS and the 24‐months estimated OS was 94% and 88% for the whole cohort. The 12‐months OS were 77%, 94% and 100% in group A, B and C, respectively (*P* = 0.12) (Figure 1). After a median follow up of 16 months, discontinuation occurred in 14 patients (14%) and the main cause of discontinuation was for adverse events (AEs) (30%), in 4% of the cohort. AE were identified 57% of the patients. Fifty‐two patients (53%) had extraematological toxicities and 17 patients (17%) had haematological toxicities. The most experienced extraematological toxicity was headache (15%) of which grade (G) 3 was observed in only 6% of cases while no G4 headache reported. Arthralgias (3%), hypertension (3%) and atrial fibrillation (1%) were found with very low incidences and always of mild grade (G1 and G2). Furthermore, we found a discrete rate of infections and skin, gastrointestinal and liver toxicities from acalabrutinib (29%). With regard to haematological toxicity, the most represented was anaemia, with a rate of 10% (Supplementary material). Our analysis confirms good efficacy of acalabrutinib in patients with HR CLL in terms of ORR, slightly higher than the ELEVATE TN trial that had a longer follow‐up of 28 months [9]. Results from this analysis of patients treated with acalabrutinib confirm durable responses, PFS and OS rates similar to patients without HR features [5, 6]. Acalabrutinib caused both haematological and extra haematological toxicities and we observed lower discontinuation rates due to AEs than those reported in randomised trials [9]. The reason for this difference could be the shorter follow‐up of our study or alternatively it could be an indication that over time we have learned to better manage toxicities and avoid suspension. Here we confirm however, as also described in the pooled analysis of HR patients treated in clinical trials with acalabrutinib, that in the first line the major cause of discontinuation of acalabrutinib is due to adverse events; However, this finding has little statistical significance given the small sample size and short follow‐up [10]. Our results also suggest that the major toxicity of acalabrutinib is the extra haematological one, as headache, compared with a mild haematological toxicity of less than 25% of cases (supplementary material). Our analysis showed good results even in terms of survival variables such as PFS and OS consistent with previous trials [9]. Furthermore, no clear efficacy or survival advantages of acalabrutinib according to a particular HR biological set‐up [del(17p) and/or *TP53*m only, uIGHV only, uIGHV with del(17p) and/or *TP53*m patients] emerged, but larger sample size and longer follow up are needed to strengthen our results. Possible limitations of this study are its retrospective nature and the relatively short median observation time of the cohort; the main strength of the study is the number of patients with HR CLL treated with acalabrutinib in real world clinical practice. In conclusion, acalabrutinib is a good option in real life in patients with HR CLL. Our study demonstrates effectiveness, long term benefits and low rate of treatment discontinuation with acalabrutinib as continuous drug in TN HR CLL population.

**TABLE 1:** Patients clinical and biological characteristics at baseline, overall response rate and causes of discontinuation.

Clinical and biological characteristics at baseline	TN CLL (*n* = 98)	*p* value
Age, years (median, IQR)	70 (65–77)	0.14
Gender, *n* (%)		
M	62 (63)	0.65
F	36 (37)	
uIGHV, *n* (%)	87 (90)	0.67
Del(17p), *n* (%)	23 (24)	0.53
*TP53*m, *n* (%)	17 (18)	
Group		
del(17p) and/or *TP53*m only	10 (10)	0.35
uIGHV only	68 (71)	
uIGHV with del(17p) and/or *TP53*m	18 (19)	
Rai stage, *n* (%)		
Low (I‐II)	44 (45)	0.36
High (III‐IV)	54 (55)	
Binet stage, *n* (%)		
A	5 (5)	0.43
B	46 (47)	
C	47 (48)	
Lymph nodes burden, *n* (%)		
No nodes	7 (7)	0.90
< 5 cm	52 (54)	
5–10 cm	27 (27)	
> 10 cm	12 (12)	
Splenomegaly, *n* (%)		
No	28 (29)	0.16
Yes	70 (71)	
Overall response rate and discontinuation		
ORR 12 months, %		
del(17p) and/or TP53m only	4 (80)	0.49
uIGHV only	54 (95)	0.78
uIGHV with del(17p) and/or TP53m	14 (93)	0.21
Patients remaining on treatment, *n* (%)	84 (86)	0.80
Temporary suspension, *n* (%)	24 (29)	0.92
Definitive treatment discontinuation, *n* (%)	14 (14)	0.81
Reason for treatment discontinuation		
Progressive disease, *n* (%)	2 (14)	
Richter transformation, *n* (%)	3 (21)	
Adverse event, *n* (%)	4 (30)	
Death, *n* (%)	3 (21)	

## Ethics Statement

This study was conducted in accordance with the Declaration of Helsinki and approved by the Institutional Review Board of Fondazione Policlinico Universitario Agostino Gemelli IRCCS.

## Consent

Written informed consent was obtained from the patients to publish this paper.

## Conflicts of Interest

The authors declare no conflicts of interest.

## Supporting information

Table S1

## Data Availability

Data available on request.
