# Supplementary material for: Acalabrutinib in High‐Risk Chronic Lymphocytic Leukaemia Naïve Patients: An Italian Multicenter Retrospective Observational Real‐Life Experience
Source: Hematol Oncol. 2025 Jan 14;43(1):e70033. doi: 10.1002/hon.70033 (PMC11732783; doi:10.1002/hon.70033)
Supplement: Supplementary file 1 — Table S1 [file HON-43-e70033-s001.docx]

|  | **TN CLL (n =98)** | **P value** |
| --- | --- | --- |
| **Extrahaematological toxicity, n (%)** | **52 (53)** | 0.42 |
| **Headache, n(%)**  **G1**  **G2**  **G3**  **Gx*** | 15 (15)  6 (6)  7 (7)  1 (1)  1 (1) |  |
| **Arthralgie, n (%)**  **G1**  **G2** | 3 (3)  1 (1)  2 (2) |  |
| **Atrial Fibrillation, n (%)**  **Gx** | 1 (1)  1 (1) |  |
| **Hypertension, n (%)**  **G2**  **G3** | 3 (3)  2 (2)  1 (1) |  |
| **Bleeding, n (%)**  **G1**  **G2**  **G3** | 12 (12)  4 (4)  3 (3)  5 (5) |  |
| **Others, n (%)** | 28 (29) |  |
| **Haematological toxicity, n (%)** | **17 (17)** | 0.51 |
| **Anemia, n (%)** | 10 (10) |  |
| **Thrombocytopenia, n (%)** | 3 (3) |  |
| **Neutropenia, n (%)** | 7 (7) |  |

*not available

**Supplemental Table 1. Adverse events**
